# Survey data on household spatial quality and experiences of stress

**DOI:** 10.1016/j.dib.2016.03.010

**Published:** 2016-03-09

**Authors:** Grace Campagna

**Affiliations:** Bronx Community College of the City University of New York, United States

## Abstract

This data article describes a dataset of 1,668 cases representing self-reported assessments of housing inadequacy and perceived housing stress. The dataset also contains person-level and household-level demographic data to contextualize the above measures. A second supplemental file contains the text of the survey instrument. Discussion of theoretical background and measures development as well as a more detailed socioeconomic profile of the sample is available in the associated research article http://dx.doi.org/10.1016/j.jenvp.2016.01.002(Campagna, 2016) [1].

**Specifications Table**

**Table:** 

Subject area	Psychology
More specific subject area	Housing quality and stress
Type of data	Excel spreadsheet; Word document of instrument
How data was acquired	Self-reported survey from a convenience sample
Data format	Raw data with some reverse-coded and computed fields
Experimental factors	All retained cases resided with other individuals and at their current dwelling for at least three months. Dwelling-attribute difference scores were computed as the gap between reported availability and importance of various spatial features.
Experimental features	Dwelling-level crowding, household composition, adequacy of dwelling spatial and psychosocial attributes, respondents’ perceived efficacy and perceived stress
Data source location	New York City, United States
Data accessibility	Data is with this article.

**Value of the data**

• The raw data of self-reported spatial attributes and psychosocial perceptions may be examined using such statistical methods as analysis of variance, linear regression, factor analysis, or structural equation modeling.
• The dataset includes person-level and household-level demographic attributes. The categorical coding of these fields may allow for comparisons of between-group differences from this sample to parallel samples in other similar studies elsewhere.
• The self-reported assessments of perceived dwelling quality are defined within a transactional model of person-environment fit. Analyses resulting from data generated by such a conceptual lens may be compared to findings from other datasets collected using alternative theoretical models of housing satisfaction.

## 1 Data

Each of the 1668 cases contains the self-reported responses of a single study participant. The data are grouped into fields that recorded individual-level attributes, dwelling-level social and spatial features, and respondents’ self-reported perceptions of stress and efficacy. Table 1 summarizes the variables contained in the provided data file.

## 2 Experimental Design, Materials and Methods

### 2.1 Experimental design

The dataset contained in the Excel file includes demographic information at the level of the individual and of the household and also self-reported responses to two scales measuring, respectively, household interior inadequacy and perceived housing stress.

Individual demographic data include respondent age, gender, hours employed per week, and several items pertaining to geographic origin. Household-level scale-type data include total household size, number of minor children up to age 17, number of habitable rooms, and number of rooms used for sleeping. Items regarding household composition asked about the presence of seven sorts of immediate family, extended kin, and persons not related to the respondent. Household composition data asked whether any children in the household fell into three specified age ranges. Respondents were also asked about their contribution to household expenses, with four options ranging from paying all accounts to not contributing at all to household expenses. As well, respondents were asked whether they provided a significant amount of caregiving for minor children or for elderly or disabled persons living in the same household.

Scale items measuring housing inadequacy focused on seven spatial attributes of the dwelling interior. Some items asked about physical features such as storage and functionality of utilities and mechanical systems, whereas other items focused on spatial features related to interpersonal interaction. Theses latter items were written to elicit information about dwelling characteristics noted as significant in the environmental psychology literature [2]. Among these desirable features are privacy [3] and spaces for retreat and restoration [4], [5]. Respondents first answered to what extent each of seven domestic interior features was available to them and then answered how important each domestic interior feature was to them.

The perceived housing stress items were adapted from the ten-item Perceived Stress Scale developed by Cohen and Williamson [6]. The original Perceived Stress Scale is a global measure whereas the rephrased items used here focused the responses on the experience of efficacy and helplessness within the household. Two additional items were added to capture the adverse effects of social withdrawal [7], [8] and the mitigating effects of predictability [9].

### 2.2 Materials

Survey data was collected via an anonymous self-administered paper questionnaire. The Word file included in the supplementary material contains the full phrasing of the survey items.

### 2.3 Method

Participants self-selected into this convenience sample. Respondents were community residents enrolled at a junior college in an outer borough of New York City, and all were students in an introductory psychology course at the time of survey administration. As part of a human-subjects approved protocol, respondents could choose among various options for fulfilling a course research participation requirement. During 2012 and 2013, a total of 1885 respondents completed the survey. The present dataset of 1668 cases did not retain 104 where the respondents reported living alone and another 113 where the respondents reported living at their home for less than three months.

For the household inadequacy assessment, the dataset contains three variables for each of the seven attributes described above under *Experimental design*. The first and second paired sets of items each used Likert-type responses [Table 1]. The first set of items asked about the availability of each feature, ranging in four intervals from *never* to *always*, whereas the second set of items asked about the importance of each dwelling feature to the respondent, ranging in four intervals from *not at all* important to *very* important. The availability features were coded to range from −3 (*never*) to 0 (*always*), and the importance features were coded to range from 0 (*not at all*) to 3 (*very*). The third set of variables represented difference scores for each corresponding pair of availability and importance items. This third set of variables was computed by subtracting the availability score from the importance score for each attribute׳s respective pair, hence *diff*1=*want*1 *– have*1. (In the provided data file, the seven computed difference scores are highlighted for clarity.) A difference score of up to three intervals indicated adequacy of the particular feature, but a difference score of greater than three intervals indicated inadequacy of the particular feature.

Scale items measuring perceived housing stress used Likert-type responses to elicit respondents’ perceptions in five possible intervals ranging from *never* to *very often*. Seven of these items measured helplessness and have negative valence, whereas five items measured efficacy and have positive valence. Of the positive-valence items, the provided dataset uses the suffix “o” after the variable label to indicate the original coding and the suffix “R” to indicate the reverse-coded data. The provided dataset highlights the reverse-coded positive-valence items. (Refer to supplementary materials.)

## Figures and Tables

**Table 1:** Variables, variable types, and value labels.

Field(s)	Variable(s)	Variable type	Value labels
f1m0	Gender	Nominal (dummy)	0: Male; 1: Female
Age	Age	Scale	As reported
WrkHrs	Hours worked per week	Scale	As reported
HHn	Household size	Scale	As reported
partner	Respondent׳s spouse/long-term partner	Nominal (dummy)	0: None in household;
1: Present in household
parent	Respondent׳s parent(s)	Nominal (dummy)	0: None in household;
1: Present in household
sib	Respondent׳s sibling(s)	Nominal (dummy)	0: None in household;
1: Present in household
ownkid	Respondent׳s minor child(ren)	Nominal (dummy)	0: None in household;
1: Present in household
grndprnt	Respondent׳s grandparent(s)	Nominal (dummy)	0: None in household;
1: Present in household
otherkin	Respondent׳s other kin	Nominal (dummy)	0: None in household;
1: Present in household
nonkin	Household member(s) unrelated to respondent	Nominal (dummy)	0: None in household;
1: Present in household
allkidsN	Total number of minors	Scale	As reported
anykid_5	Children up to age 5	Nominal (dummy)	0: None in household;
1: Present in household
anykid6_12	Children ages 6 to 12	Nominal (dummy)	0: None in household;
1: Present in household
anyteen	Teens ages 13 to 17	Nominal (dummy)	0: None in household;
1: Present in household
carechild	Significant caregiving for child(ren)	Nominal (dummy)	0: Not applicable;
1: Applicable
careelder	Significant caregiving for elderly or disabled	Nominal (dummy)	0: Not applicable;
1: Applicable
rms	Number of habitable rooms	Scale	As reported
brms	Number of rooms used for sleeping	Scale	As reported
hhcosts	Respondent׳s dwelling payment burden	Ordinal	1: Pays all costs;
2: Pays most costs and others contribute;
3: Contributes to others’ payments;
4: No share of costs
imm	Time since immigration	Categorical	1: Born and raised in USA;
2: Immigrated over 10 years ago
3: Immigrated five to 10 years ago;
4: Immigrated one to five years ago;
5: Immigrated during past year
fromarea	Geographic origin (where respondent grew up)	Categorical	1: Northeastern USA;
2: Southern USA;
3: Other USA;
4: Central/South America;
5: Caribbean;
6: Sub-Saharan Africa;
7: North Africa/Middle East;
8: Europe
9: Other area
areasize	Type of community of origin	Ordinal	1: Large city;
2: Medium-size town
3: Small town
4: Rural area
have1… have7 a	(availability of various dwelling spatial features)	Ordinal	0: All of the time;
−1: Most of the time;
−2: Some of the time;
−3: Never
want1… want7 b	(reported importance of various dwelling spatial features)	Ordinal	0: Not at all;
1: A little;
2: Somewhat;
3: Very
PHS_1… PHS_12 c	(respondent׳s perceptions of own dwelling-related stress and efficacy)	Ordinal	1: Never;
2: Almost never;
3: Sometimes;
4: Fairly often;
5: Very often

a For the inventory of each of the seven availability items, refer to supplementary material.

b For the inventory of each of the seven importance items, refer to supplementary material.

c For phrasing of each of the twelve perceived stress items, refer to supplementary material.
