# Supplementary material for: Survey data on household spatial quality and experiences of stress
Source: Data Brief. 2016 Mar 9;7:509–13. doi: 10.1016/j.dib.2016.03.010 (PMC4796714; doi:10.1016/j.dib.2016.03.010)
Supplement: Supplementary file 2 — Supplementary material [file mmc2.zip › DIB-D-16-00025 supplementary material/DIB-D-16-00025 Supplementary material_instrument.doc]

**Questionnaire**

**Section I**

1. What is your gender?	_____ Male	_____ Female

2. How old are you?	________

3. Do you have a steady job right now?	_____ No	_____ Yes

If YES, how many hours do you usually work per week?	__________

*The following questions are about where you now live. Your “household” is an apartment if you live in an apartment, or a house if you live in a house.*

4. Do you live alone or with others? _____ Alone (*Please continue on the next side >>>>)*

_____ With others *(Please answer the rest of this page first.)*

5. Including yourself, how many people live in your household? ______

*(Please count anyone who stays there more than they stay anyplace else.)*

6. How are these people connected to you? *Check as many as apply*.

_____ my spouse/long-term partner	_____ my grandparent(s)

_____ my parent(s)	_____ my other relatives (such as aunt, uncle, cousin,

_____ my sibling(s) (brother/sister)	stepchild, niece, nephew, grandchild, etc.)

_____ my child / children	_____ someone not related to anyone in the household

7. Are there children under age 18 in your household? _____ No	_____ Yes

*If yes, how many?* ________

*Please check (√) their age ranges:* _____ up to age 5	_____ ages 6-12	_____ ages 13-17

8a. “I spend a lot of time taking care of one or more of the children who live with me.” (Please √ )

_____ very true	_____ a little true	_____ not at all

8b. “I spend a lot of time taking care of an elderly or disabled adult who lives with me.” (Please √ )

_____ very true	_____ a little true	_____ not at all

9. Which is the closest way to describe your monthly housing costs (rent, electricity, etc.) *Check one*:

_____ I pay all the household monthly costs.

_____ Everything (or almost) is in my name, but the people I live with give me some money.

_____ Everything (or almost) is in someone else’s name, and I give them some money.

_____ I don’t contribute any money for my monthly housing costs.

10. Not counting hallways and bathrooms, how many rooms are in your home/apartment? ______ *(Count the kitchen only if it is big enough for a table and chairs, and people can sit there.)*

11. How many of these rooms usually have somebody sleeping there at night?	______

12. Which of the following statements best describes your geographic background?

*(If you were born in the US but grew up elsewhere, please answer based on how long it has been since you returned.)*

_____ I was born and grew up only in the USA.

_____ I was born outside the USA and moved to this country over 10 years ago.

_____ I was born outside the USA and moved to this country between 5 and 10 years ago.

_____ I was born outside the USA and moved to this country between 1 and 5 years ago.

_____ I was born outside the USA and moved to this country in the past 12 months.

13. In which one of the following places did you spend MOST of your childhood (up to age 17)?

_______ Northeastern USA	_______ Africa (south of the Sahara)

_______ Southern part of the USA	_______ Middle East or North Africa

_______ Some other part of the USA	_______ Europe

_______ Central America or South America	_______ East Asia or a Pacific Island

_______ One of the Caribbean islands	_______ South or Central Asia

14. Was the place where you spent most or all of your childhood a:

_____ large city	_____ village or small town where most people knew each other

_____ medium-sized city or town	_____ rural area where the houses were far apart.

**Section II**

*For Questions 1 through 7*, *please think about the housing you live in now, and indicate whether each of these situations, items, etc. is AVAILABLE to you.*

*“Right now, I have …:*

1) an interior (walls, floors, furniture, etc.) that looks good.

_____ all of the time	_____ most of the time	_____ some of the time	_____ never

2) enough comfortable space inside the home to invite friends or relatives to visit.

_____ all of the time	_____ most of the time	_____ some of the time	_____ never

3) plumbing, electricity, and heat that work the way that they are supposed to.

_____ all of the time	_____ most of the time	_____ some of the time	_____ never

4) enough places inside the household for storage, to put things away easily.

_____ all of the time	_____ most of the time	_____ some of the time	_____ never

5) a place inside my home where everybody who lives there can be together comfortably.

_____ all of the time	_____ most of the time	_____ some of the time	_____ never

6) a place inside the household where some of the people can have privacy together apart from others in the home.

_____ all of the time	_____ most of the time	_____ some of the time	_____ never

7) a place inside my home where I can spend quiet time alone whenever I want to.

_____ all of the time	_____ most of the time	_____ some of the time	_____ never

*For Questions 8 through 14, please indicate how IMPORTANT to you each of these situations or things are to you , whether or not it’s actually available to you in the housing you have right now.*

*“Right now, it’s important for me to have …:*

8) an interior (walls, floors, furniture, etc.) that looks good.

_____ very important	_____ a little important	_____ not too important	_____ not at all important

9) enough comfortable space inside the home to invite friends or relatives to visit.

_____ very important	_____ a little important	_____ not too important	_____ not at all important

10) plumbing, electricity, and heat that work the way that they are supposed to.

_____ very important	_____ a little important	_____ not too important	_____ not at all important

11) enough places inside the household for storage, to put things away easily.

_____ very important	_____ a little important	_____ not too important	_____ not at all important

12) a place inside my home where everybody who lives there can be together comfortably.

_____ very important	_____ a little important	_____ not too important	_____ not at all important

13) a place inside the household where some of the people can have privacy together apart from others in the home.

_____ very important	_____ a little important	_____ not too important	_____ not at all important

14) a place inside my home where I can spend quiet time alone whenever I want to.

_____ very important	_____ a little important	_____ not too important	_____ not at all important

15) How long have you lived in the home that you’re answering these questions about?

____ under a month	____ 1 to 3 months	____ 4 to 6 months	____ 7 to 12 months	____ over a year

**Section III**

*The questions in this section ask you about your feelings and thoughts during the last month. In each case, please check* (*√ ) how often you felt or thought a certain way.*

1. How often have you been upset because of some unexpected problem with the physical condition of your home?

*_____ never	_____ almost never	_____ sometimes	_____ fairly often	_____ very often*

2. How often have you felt that you couldn’t do anything about some physical condition inside your home?

*_____ never	_____ almost never	_____ sometimes	_____ fairly often	_____ very often*

3. How often have you felt nervous and stressed by how crowded your home is?

*_____ never	_____ almost never	_____ sometimes	_____ fairly often	_____ very often*

4. How often have you felt confident about your ability to deal with noise or other irritating distraction happening inside your home?

*_____ never	_____ almost never	_____ sometimes	_____ fairly often	_____ very often*

5. How often have you felt satisfied that things were going your way in how you use the space inside your home?

*_____ never	_____ almost never	_____ sometimes	_____ fairly often	_____ very often*

6. How often have you felt that you could not manage everything you had to do to take care of the place where you live?

*_____ never	_____ almost never	_____ sometimes	_____ fairly often	_____ very often*

7*.* How often have you felt confident about your ability to deal with noise or other irritating distractions from neighbors in your building or from nearby buildings?

*_____ never	_____ almost never	_____ sometimes	_____ fairly often	_____ very often*

8. How often have you felt that you were able to keep the places in your home comfortable enough for yourself?

*_____ never	_____ almost never	_____ sometimes	_____ fairly often	_____ very often*

9. How often have you been upset that you didn’t have enough privacy or personal “space” for yourself inside your home?

*_____ never	_____ almost never	_____ sometimes	_____ fairly often	_____ very often*

10. How often have you felt that your responsibilities at home were piling up so high that you could not overcome them?

*_____ never	_____ almost never	_____ sometimes	_____ fairly often	_____ very often*

11. How often have you found yourself withdrawing from or trying to ignore what was going at home?

*_____ never	_____ almost never	_____ sometimes	_____ fairly often	_____ very often*

12. How often have you felt comfortable that could predict how things would happen at home each day?

*_____ never	_____ almost never	_____ sometimes	_____ fairly often	_____ very often*
